# A comprehensive analysis of the tryptophan metabolism-related gene signature to predict the prognosis of esophageal squamous cell carcinoma based on multi-omics

**DOI:** 10.3389/fmolb.2025.1613539

**Published:** 2025-08-01

**Authors:** Zhengjie Wu, Zhiping Liu, Yukun Wang, Geling Teng, Xiaodong Li, Tong Lu, Fangning Hu, Shuo Wu, Guanqiang Ma, Hua Zhang

**Affiliations:** ^1^ Tracheal and Bronchial Surgery, Shandong Public Health Clinical Center, Shandong University, Jinan, China; ^2^ Department of Oncology, Shandong Provincial Third Hospital, Shandong University, Jinan, China; ^3^ Department of Geriatrics, Affiliated Hospital of Qingdao BinHai University, Qingdao, China; ^4^ Department of Respiratory and Critical Care Medicine, Shandong Public Health Clinical Center, Shandong University, Jinan, China; ^5^ Department of Thoracic Surgery, Shandong Public Health Clinical Center, Shandong University, Jinan, China

**Keywords:** tryptophan metabolism, esophageal squamous cell carcinoma, multi-omics, tumor environment, prognosis

## Abstract

**Background:**

Tryptophan (Trp) metabolism plays a vital role in tumor development and outcomes. However, Trp in esophageal squamous cell carcinoma (ESCC) remains poorly understood. We aimed to explore the role and mechanism of Trp metabolism in ESCC.

**Methods:**

We integrated single-cell RNA (scRNA) sequencing, bulk transcriptome, proteomics, and microbiome data from public databases. Tryptophan-related cell populations and their interactions were explored using the “seurat” R package at the single-cell level. Least absolute shrinkage and selection operator (LASSO) and univariate Cox regression were used to select prognostic TrpGs and construct a risk model. The overall survival, immune infiltration, checkpoint expression, drug sensitivity, and microbiota composition between high- and low-risk groups were evaluated. Independent prognostic factors were identified via multivariate Cox analysis and validated by qPCR analysis, and a nomogram was constructed for survival prediction.

**Results:**

We identified 28 differentially expressed tryptophan-related genes (DE-TrpGs), and fibroblasts emerged as the cell type with the highest TrpG score, although reduced in ESCC. Eighteen DE-TrpGs showed downregulation in tumor fibroblasts at the single-cell level. Fibroblast-epithelial communication involved the LAMININ, HSPG, and AGRN pathways. Five prognostic TrpGs (MAOA, AKR1A1, ALDH9A1, HAAO, and ALDH2) were selected to construct the risk model. The expression of MAOA, AKR1A1, ALDH9A1, HAAO, and ALDH2 was significantly downregulated in ESCC tumor tissues compared to non-tumor tissues. High-risk patients showed poorer overall survival (OS), distinct immune cell infiltration, elevated expression of KIR2DL1, LGALS9, TNFRSF18, and TNFRSF4, increased sensitivity to imatinib and axitinib, resistance to multiple chemotherapeutics, and reduced *Fusobacteria* and *Tenericutes* abundance. HAAO, ALDH2, and lymph node stage were identified as independent prognostic factors and were used to develop a predictive nomogram.

**Conclusion:**

We identified a Trp metabolism-associated fibroblast population in the ESCC tumor microenvironment (TME) and developed a five-gene TrpG signature for prognostic prediction in ESCC patients.

## 1 Introduction

Esophageal cancer (ESC) is a common cancer, ranking seventh in terms of incidence and sixth in mortality among all cancer types (Sung et al., 2021). Overall, incidence and mortality rates are two- to three-fold higher in men than in women (Morgan et al., 2022). Regions with high incidence rates include Eastern Asia, along with Southern Africa, Eastern Africa, Northern Europe, and South Central Asia (Abnet et al., 2018). Esophageal squamous cell carcinoma (ESCC) and adenocarcinoma (EADC) are the two most common histologic subtypes. Smoking and heavy drinking are the major risk factors for ESCC in Western countries; however, the major risk factors are different in developing countries, and dietary components are suspected risk factors (Armstrong, 2018; McCorm et al., 2017). ESCC is characterized by late-stage diagnosis, metastasis, therapy resistance, and frequent recurrence (Morgan et al., 2022; Reichenbach et al., 2019). Survival of ESCC remains low, with an appropriate range of 10%–30% at 5 years post-diagnosis in the majority of countries (Allemani et al., 2018). Clinical management of ESCC includes endoscopic resection, surgical resection, radiochemotherapy, neochemotherapy, and adjuvant immunotherapy (Puhr et al., 2023). However, clinical outcomes remain poor, with limited efficacy, severe side effects, and heterogeneity (Yang et al., 2020). Thus, understanding the molecular characteristics of ESCC helps explore efficiency biomarkers and personalized therapeutic options.

Metabolism reprogramming is a hallmark of malignancy and plays a pivotal role in tumorigenesis, development, metastasis, therapy resistance, and recurrence (Faubert et al., 2020). Targeting metabolic characteristics is a crucial opportunity for cancer therapy. However, the majority of metabolic biomarkers for cancer therapy remain unclear. Tryptophan (Trp) is an essential amino acid for humans. Most tryptophan is metabolized through the kynurenine (Kyn) pathway (Xue C. et al., 2023), and Trp metabolism is involved in the regulation of immune and energy balance in cancer (Molfino et al., 2024; Seo and Kwon, 2023). Notably, Trp metabolism promotes macrophage M1 polarization and serves as a promising predictor for the effectiveness of immunotherapy in breast cancer (Xue L. et al., 2023). Rate-limiting enzymes indoleamine-2,3-dioxygenase 1 (IDO1), IDO2, tryptophan-2,3-dioxygenase (TDO), and kynurenine monooxygenase (KMO) initiate the Kyn pathway, converting Trp into L-kynurenine (Kyn) and other downstream metabolites (Platten et al., 2019). IOD1 and TDO have been implicated in cancer-related immunosuppression, and their inhibition can alleviate this immunosuppressive effect (Liang et al., 2021; Du et al., 2020). These findings collectively highlight Trp metabolism as a key regulator of immune evasion in cancer. Serum metabolomics analyses have revealed that elevated serum L-tryptophan levels are associated with a reduced risk of developing ESCC (Li X. et al., 2021). Additionally, other metabolomic studies have demonstrated that dysregulated Trp metabolism contributes to the progression and metastasis of ESCC (Cheng et al., 2017; Chen et al., 2022). However, the regulatory mechanisms of Trp metabolism within the tumor microenvironment (TME) of ESCC remain largely unknown. Given the critical role of Trp metabolism in tumorigenesis and cancer progression, a comprehensive analysis of its key molecular biomarkers is warranted to identify novel therapeutic targets for ESCC.

In the present study, we comprehensively explored the Trp-related gene (TrpG) signature and specific cell types in ESCC based on single-cell RNA-sequencing data, transcriptome data, proteomics data, and microbial abundance profiles. We further evaluated and experimentally validated the clinical significance of the TrpG signature, as well as its immune characteristics, drug sensitivity, and microbial composition. Our findings highlight potential biomarkers and therapeutic targets for interventions aimed at modulating tryptophan metabolism in ESCC.

## 2 Materials and methods

### 2.1 Data collection and processing

Gene expression profiles and corresponding clinical features of ESCC patients were obtained from the GSE53625 (179 tumor tissues and 179 normal tissues) and GSE121931 (125 tumor tissues) cohorts from the Gene Expression Omnibus (GEO) database. Raw proteomics data and corresponding clinical features from PXD021701 (124 ESCC tissues and 124 normal tissues) were obtained from the PRoteomics ID Entifications Database (PRIDE Archive). ESCC scRNA-seq data were acquired from GSE197677 (10 solid tumor tissues and four normal tissues). Microbial abundance profiles at different taxonomic levels were obtained from the Cancer Microbiome Atlas (TCMA, https://tcma.pratt.duke.edu/) database for 40 ESCC tumor samples. A total of 66 tryptophan metabolism-related genes (TrpGs) were obtained from the Human Gene Set “KEGG_TRYPTOPHAN_METABOLISM” in the Molecular Signatures Database (MSigDB, https://www.gsea-msigdb.org/gsea/msigdb), and from the “superpathway of tryptophan utilization” in PathCards (https://pathcards.genecards.org/Card/superpathway_of_tryptophan_utilization). The TrpGs are listed in Supplementary Table S1.

### 2.2 Identification of the differentially expressed TrpGs in ESCC

Differentially expressed genes (DEGs) between ESCC tumor tissues and normal tissues (GSE53625 cohort) were identified using the “Limma” R package with the criteria of |log(Fold change, FC)| > 0.5 and P-value < 0.01. Then, the differentially expressed TrpGs (DE-TrpGs) were selected by overlapping the DEGs in ESCC and TrpGs from the public databases.

### 2.3 Single-cell RNA sequencing data processing, cell annotation, and calculation of TrpG score

The scRNA-seq data were processed and assessed using the “Seurat” R package. Quality control, normalization, and clustering were finished before data acquisition (Okuda et al., 2023). Therefore, a total of 29,473 genes and 67,626 cells, and an expression matrix were included for subsequent analyses in this study. Cells were annotated using known cell marker genes. The “COSG” R package was used to implement Gene Ontology (GO) and Kyoto Encyclopedia of Genes and Genomes (KEGG) enrichment analyses for each cell type, based on the top 2,000 marker genes in each cell type. We also detected the expression of DE-TrpGs in each cell population. The TrpG score for each cell population was calculated using the “UCell” R package based on 66 TrpGs. The cell population with the highest TrpG score was selected for further analysis.

### 2.4 Intercellular communication analysis

The “CopyCAT” R package was used to analyze the copy number variant (CNV) characteristics of the epithelial cell populations and distinguish tumor cells from normal ones. The “COSG” R package was used for GO enrichment analysis based on the marker genes. Then, the “CellChat” R package was used to investigate the potential interactions between tumor cells and high-TrpG score cells. The CellChat analysis was complementarily used for cell communication analysis based on the CellChat DB, which contains 2,021 validated interactions, including 60% of paracrine/autocrine signaling interactions, 21% of extracellular matrix (ECM)–receptor interactions, and 19% of cell–cell contact interactions (Jin et al., 2021).

### 2.5 Construction and validation of a TrpG risk model

The least absolute shrinkage and selection operator machine learning algorithm (LASSO) regression was used to select the prognostic TrpGs in the GSE121931 cohort using the “glmnet” R package. Univariate Cox regression analysis was used to screen protective factors for ESCC among the above LASSO-generated risk factors in the GSE121931 and PXD021701 cohorts. The hazard ratio (HR) and 95% confidence intervals (CI) were calculated. TrpGs with HR > 1 were identified as risk factors, and those with HR < 1 were identified as protective factors. A bilateral p < 0.05 was considered statistically significant. Based on these protective factors, the “ggrisk” R package was used to calculate a risk score and construct a TrpG risk model in the GSE121931 cohort. The ESCC patients were distributed into high-risk score and low-risk score groups based on the median value of the risk score. Kaplan–Meier overall survival (OS) curves were drawn using the “survival” R package for the training (GSE121931) and validation (PXD021701) cohorts. Receiver operating characteristic (ROC) curves for 1-, 2-, and 3-year survival probabilities for ESCC patients were drawn using the “timeROC” R package. Then, a nomogram was established using the “rms” R package according to independent prognostic factors. The calibration curves and decision curves were drawn to verify the accuracy of the nomogram.

### 2.6 Analysis of the immune cell infiltration landscape in TrpG risk score groups

The marker gene set of 28 immune cells was obtained and referenced from a previous article (Charoentong et al., 2017). The “IOBR” R package was used to estimate the proportion of immune cells in the high- and low-risk groups. The correlation between infiltrated cells and risk scores was also assessed by the “IOBR” R package. The immune score, stromal score, and ESTIMATE score were calculated using the ESTIMATE algorithm in the “IOBR” R package. The differences in each score between the high- and low-risk groups were determined by Student’s t-test, ***p < 0.001, **p < 0.01, and *p < 0.05. The differences in the expression of immune checkpoint genes between the high- and low-risk groups were determined by Student’s t-test (***p < 0.001, **p < 0.01, and *p < 0.05), and the correlation between the expression of immune checkpoint genes and risk scores was detected using a Spearman’s correlation analysis.

### 2.7 Drug sensitivity analysis of ESCC patients in different TrpG risk score groups

The NCI-60 compound activity data and RNA-seq expression profiles from the CallMiner™ (https://discover.nci.nih.gov/cellminer/home.do) were obtained to investigate the drug sensitivity. Drugs approved by the Food and Drug Administration (FDA) or in clinical trials were selected for analysis. The “pRRophetic” R package and ridge regression analysis were used to predict and evaluate the half maximal inhibitory concentration (IC50) value for each sample. The differences in IC50 value between the high- and low-risk groups were detected by the Student’s t-test (***p < 0.001, **p < 0.01, and *p < 0.05).

### 2.8 Identification of the TrpG risk score associated with microbiota landscapes

The microbial abundance profiles at different taxonomic levels for 40 ESCC tumor samples were obtained from the TCMA database. The differences in microbiota were assessed using a Student’s t-test (***p < 0.001, **p < 0.01, and *p < 0.05).

### 2.9 Specimen collection

A total of 10 ESCC tumor samples and 10 normal samples were obtained from patients who had been diagnosed with ESCC and had not received any preoperative treatment at the Shandong Public Health Clinical Center. The protocol for collecting clinical samples was approved by the Ethics Committee of the Shandong Public Health Clinical Center (approval number: 2021 XKYYEC-37), and the patients provided informed consent before samples were collected. The patients underwent surgical resection, and the samples were frozen in liquid nitrogen immediately after collection and stored at −80°C for further RNA extraction.

### 2.10 RNA extraction and quantitative real-time polymerase chain reaction (qPCR) analysis

TRIzol reagent (Takara, Dalian, China) was used to isolate the total RNA from tissues, following the standard protocol. RNA quantification was performed with a Nanodrop (Thermo Fisher Scientific, MA, United States). To compare expression levels between two groups, quantitative real-time PCR (qRT-PCR) was performed using the RNA-direct SYBR Realtime PCR Master Mix (Takara) and a Stratagene Mx3000P Real-Time PCR System (Agilent Technologies, CA, United States). The expression of each gene was determined by using GAPDH as the reference gene. Each experiment was performed in triplicate to obtain the average value. The qRT-PCR results were analyzed with the 2^−ΔΔCt^ method. The primers used are: GAPDH: CCTCAACTACATGGCTGAGAAC and CAAGGGGTCTACATGGCAACT, MAOA: CCAGCGGTAGAAATCACCCA and TCTGATGAGCACATACACGTTACTT, AKR1A1: GGGTACCTGGAAGAGTGAGC and GATCTGAGCTGGAGATCGGC, ALDH9A1: CAACCGGCCGAGTGATAGC and TGTGGTCGGTTGATGAGTGG, HAAO: AACAAGCTCATGCACCAGGA and CATGGTGTCGCCCACATAGT, ALDH2: TAATCCAGGTTGCTGCTGGG and AGTGCTCACCTCCTCCTTG.

### 2.11 Statistical analysis

All experimental results are expressed as the mean ± standard deviation (SD). Statistical analyses were performed using GraphPad Prism software, version 8.3.0 (GraphPad Software, CA, United States). The significant difference was determined using a two-tailed Student’s t-test (two-group comparisons). *P* < 0.05 indicated statistical significance.

## 3 Results

### 3.1 Identification of the DE-TrpGs in ESCC

**FIGURE 1:**
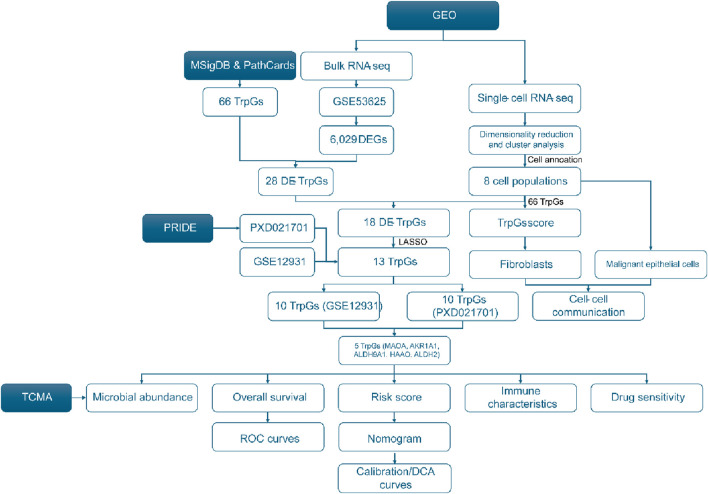
Flowchart of this study.

**FIGURE 2:**
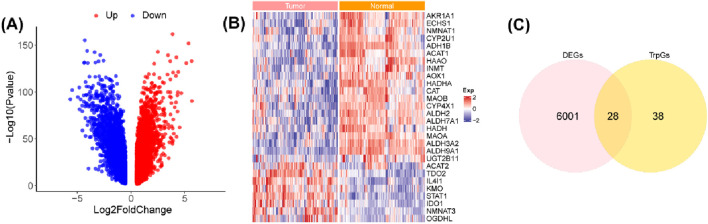
Identification of the DE-TrpGs in ESCC. **(A)** Volcano plot of the DEGs between ESCC tumor samples and normal samples with the criterion of |log(FC)| > 2 and p < 0.01. Red plots represent upregulated DEGs, and blue plots represent downregulated DEGs. **(B)** The heatmap shows the 28 DE-TrpGs in ESCC. **(C)** The Venn plot shows the 28 DE-TrpGs identified by overlapping DEGs and TrpGs.

The workflow of this study is shown in Figure 1. A total of 6,029 DEGs (3,074 upregulated and 2,955 downregulated) between ESCC samples and normal samples in the GSE53625 cohort were identified with the criterion of |log(FC)| > 2 and p < 0.01 (Figure 2A; Supplementary Table S2). Then, 28 DE-TrpGs were obtained by overlapping the 6,029 DEGs and 66 TrpGs (Figures 2B, C), which contained eight upregulated (STAT1, IL4I1, TDO2, NMNAT3, KMO, IDO1, OGDHL, and ACAT2) and 20 downregulated DE-TrpGs (ALDH9A1, ALDH3A2, ADH1B, AOX1, MAOA, ECHS1, ALDH2, ACAT1, HAAO, ALDH7A1, HADHA, MAOB, AKR1A1, CYP4X1, INMT, CYP2U1, CAT, HADH, NMNAT1, and UGT2B11) between ESCC and normal samples (Figure 2B). These results indicate that tryptophan metabolism is potentially involved in the ESCC tumorigenesis and progression.

### 3.2 Identification of eight cell populations in ESCC and calculation of TrpG score

**FIGURE 3:**
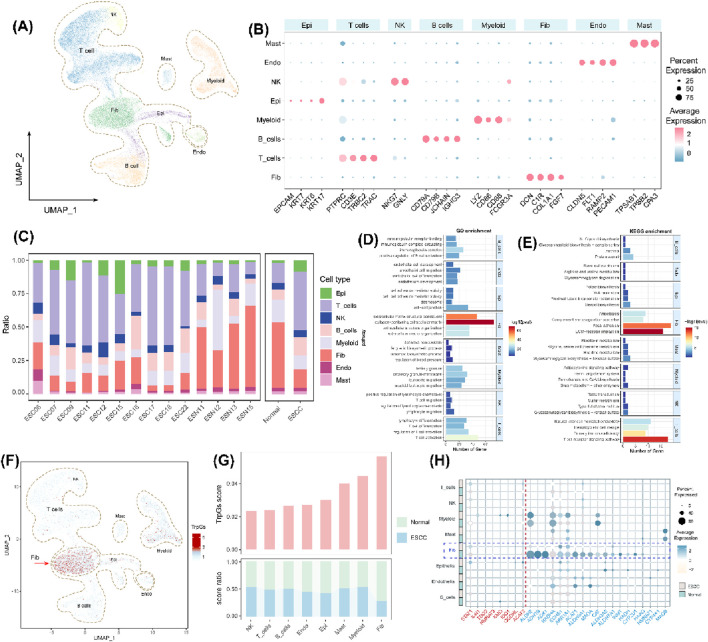
Identification of eight cell populations in ESCC and calculation of TrpG scores. **(A)** UMAP plots of the distribution of the eight cell populations based on marker gene expression. **(B)** Bubble plots of the top four marker gene expressions of each cell cluster. **(C)** Histogram of the cell numbers of each cell cluster in the ESCC samples and the normal samples. **(D, E)** Histograms of the top four GO and KEGG enrichment findings for each cell population. **(F)** UMAP plots of the distribution of the eight cell populations with different TrpG scores. **(G)** Histogram of the cell numbers of each cell cluster with different TrpG scores in the ESCC samples and the normal samples. **(H)** Bubble plots of the 28 DE-TrpG expressions of each cell cluster.

We further investigated the influence of tryptophan metabolism in the tumor microenvironment (TME) of ESCC based on single-cell gene expression profiles. Eight primary cell populations were identified with marker genes in ESCC (Figures 3A, B), including 25,627 T cells, 1,477 mast cells (Mast), 3,324 natural killer (NK) cells, 7,998 myeloid cells (Myeloid), 14,425 fibroblasts (Fib), 1,574 endothelial cells (Endo), 8,344 B cells, and 4,857 epithelial cells (Epi). We also found an increased abundance of immune cells (T cells and B cells) and epithelial cells, and a decreased abundance of fibroblasts in ESCC tumor samples compared with normal samples (Figure 3C). GO and KEGG enrichment analysis illustrated the different biological functions in the different cell populations (Figures 3D, E). Especially, extracellular matrix structural constituents, collagen-containing extracellular matrix, focal adhesion, and ECM-receptor interaction were significantly enriched in fibroblasts. The T cell receptor signaling pathway was significantly enriched in the T cell population. We also found the fibroblast population with the highest TrpG score in the tumor microenvironment (TME) of ESCC (Figure 3F), and the TrpG score in ESCC tumor samples was lower than in normal samples (Figure 3G). We next explored the expression of 28 DE-TrpGs, from which bulk RNA-seq data analysis in different cell types showed that 18 DE-TrpGs (ALDH9A1, ALDH3A2, ADH1B, AOX1, MAOA, ECHS1, ALDH2, ACAT1, HAAO, ALDH7A1, HADHA, AKR1A1, CYP4X1, INMT, CYP2U1, CAT, HADH, and NMNAT1) were significantly expressed at the single-cell level, especially the downregulated expression of fibroblasts in ESCC tumor samples compared with normal samples (Figure 3H). The single-cell RNA-seq results were consistent with the bulk RNA-seq results: 18 DE-TrpGs were downregulated in ESCC tumor samples compared with normal samples and were clearly observed in fibroblasts.

### 3.3 Intercellular communication analysis of the fibroblasts and tumor cells

**FIGURE 4:**
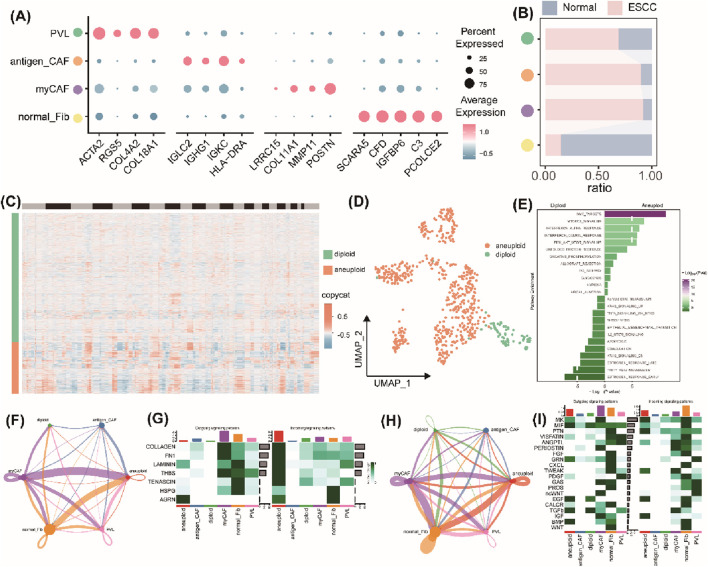
Intercellular communication analysis of the fibroblasts and tumor cells. **(A)** Bubble plots of the top four marker gene expressions of each subcluster of fibroblasts. **(B)** Histogram of the cell numbers of each subcluster of fibroblasts in the ESCC samples and the normal samples. **(C)** Heatmap of the CNV patterns in epithelial cells across the 10 ESCC samples. **(D)** UMAP plots of the distribution of malignant epithelial cells (aneuploid) and benign epithelial cells (diploid). **(E)** Histograms of the significant pathway enrichments for malignant epithelial cells and benign epithelial cells. **(F)** Cell interaction numbers and strength by ECM-receptor analysis. **(G)** A heatmap showing possible afferent or efferent signaling pathways between cells by ECM-receptor analysis. **(H)** Cell interaction numbers and strength by secreted signaling analysis. **(I)** Heatmap shows the possible afferent or efferent signaling pathways between cells by secreted signaling analysis.

The TrpG scores of fibroblasts were found to be significantly different between the normal groups and the ESCC group. Therefore, we focused on the fibroblast subtypes in ESCC. As shown in Figure 4A, four subtypes of fibroblasts were identified, including normal fibroblasts (normal_Fib), myofibroblasts (myCAF), antigen_CAFs, and perivascular‐like fibroblasts (PVL). The subtypes of normal_Fib were mainly enriched in normal samples, and the others were significantly enriched in ESCC samples (Figure 4B). Furthermore, 564 malignant epithelial cells (aneuploid) and 83 benign epithelial cells (diploid) were identified by calculating the CNV using the “copyCAT” R package (Figures 4C, D). Biological functional enrichment analysis indicated that malignant epithelial cells were significantly enriched in MYC targets, MTORC1 signaling, interferon alpha/gamma response, and PI3K/AKT/MTOR signaling pathways (Figure 4E).

Then, we created a communication network to illustrate the communication between fibroblasts and epithelial cells. ECM–receptor analysis results indicated that malignant epithelial cells mainly interacted with normal fibroblasts and myofibroblasts, but a weaker association was found between benign epithelial cells and myofibroblasts (Figure 4F). Heatmaps displayed the normal fibroblasts and myCAF, and malignant epithelial cells represented the main signaling providers and the receptors, respectively (Figure 4G). The intercellular interactions were mainly active in signaling pathways, including pathways involving LAMNIN, HSPG, and ARGN (Figure 4G). Furthermore, secreted signaling analysis results indicated a strong interaction between malignant epithelial cells and normal fibroblasts and myofibroblasts and a weaker interaction between benign epithelial cells and myofibroblasts (Figure 4H). Heatmaps also represented normal fibroblasts or myofibroblasts as the main signaling providers, and the malignant epithelial cells were represented as the main receptors (Figure 4I). The intercellular interactions were mainly involved in activation of several signaling pathways, including MK, PTN, EGF, and IGF (Figure 4I). Taken together, a strong interaction between malignant epithelial cells and normal fibroblasts or myofibroblasts and a weak interaction between benign epithelial cells and fibroblasts or myofibroblasts indicate that communication between malignant epithelial cells and normal fibroblasts or myofibroblasts is transformed in the TEM of ESCC.

### 3.4 Identification and validation of the prognostic TrpGs in ESCC

**FIGURE 5:**
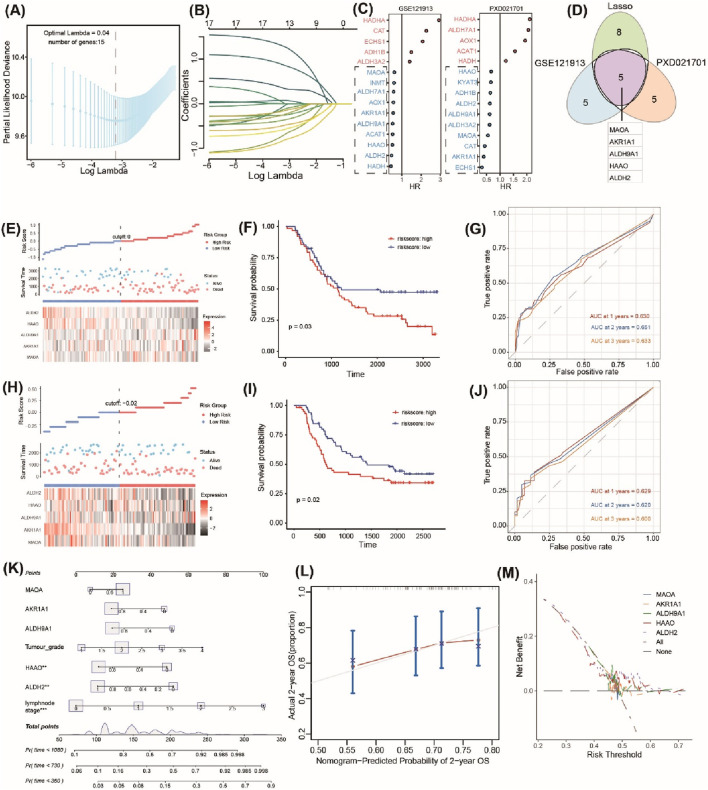
Identification and validation of the prognostic TrpGs in ESCC. **(A)** Confidence interval under lambda. **(B)** The trajectory of each independent variable with lambda. **(C)** Forest plots show the risk factors by univariate Cox analysis. **(D)** Venn plots of the signature genes by comparing the LASSO regression results and the univariate Cox results from transcriptome and protein data. **(E, H)** Distribution of TrpG risk score, survival status, and survival time in ESCC patients, and a heatmap of the five TrpGs in GSE121913 and PXD021701 datasets, respectively. **(F, I)** Kaplan–Meier curves for OS in ESCC patients with a TrpG risk score in the GSE121913 and PXD021701 datasets. **(G, J)** ROC curves for 1-, 2-, and 3-year survival rates for ESCC patients. **(K)** A nomogram prediction combining clinical characteristics and TrpG signature. **(L)** Predicted and actual 2-year survival in calibration plots for the training cohorts. **(M)** The DCA curve shows the sensitivity of the prediction nomogram.

Based on bulk RNA-seq data and scRNA-seq data analyses, 18 DE-TrpGs (ALDH9A1, ALDH3A2, ADH1B, AOX1, MAOA, ECHS1, ALDH2, ACAT1, HAAO, ALDH7A1, HADHA, AKR1A1, CYP4X1, INMT, CYP2U1, CAT, HADH, and NMNAT1) were screened in ESCC, then incorporated into a LASSO regression model to select the diagnostic TrpGs in ESCC. As a result, 13 TrpGs (ALDH9A1, ADH1B, AOX1, MAOA, ECHS1, ALDH2, ACAT1, HAAO, ALDH7A1, HADHA, AKR1A1, CAT, and HADH) were identified in the GSE121931 cohort (Figures 5A, B). In addition, univariate Cox analysis was performed to identify the protective factors for ESCC: 10 protective factors (MAOA, INMT, ALDH7A1, AOX1, AKR1A1, ALDH9A1, ACAT1, HAAO, ALDH2, and HADH) were identified in the GSE121931 cohort (Figure 5C). Ten protective factors (HAAO, KYAT3, ADH1B, ALDH2, ALDH9A1, ALDH9A2, MAOA, CAT AKR1A1, ECHS1) were identified in the PXD021701 cohort (Figure 5C). Finally, five protective factors (MAOA, AKR1A1, ALDH9A1, HAAO, and ALDH2) were obtained by overlapping the above results (Figure 5D).

**FIGURE 6:**
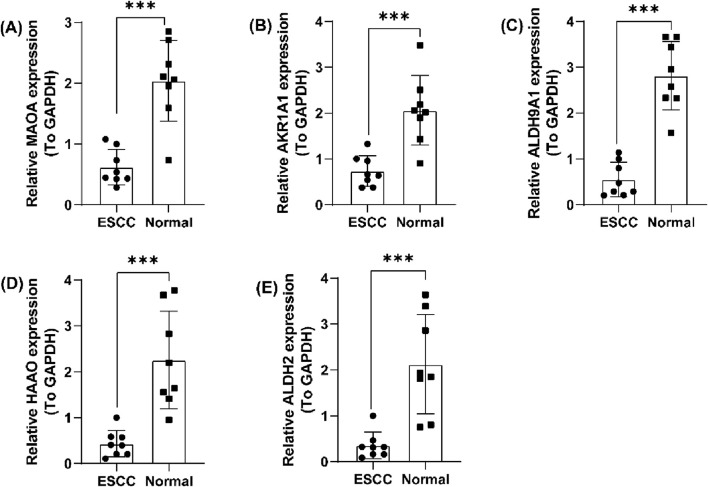
Validation of the five prognostic TrpGs in ESCC. **(A–E)** Histogram plots showing the expression of **(A)** MAOA; **(B)** AKR1A1; **(C)** ALDH9A1; **(D)** HAAO; **(E)** ALDH2 in ESCC tumor and non-tumor normal samples.

A TrpG risk score was calculated, and the patients were distributed into high-risk and low-risk groups based on the median risk score in both the GSE121931 and PXD021701 cohorts (Figures 5E, H). Kaplan–Meier OS curves indicated that patients with high-risk scores have a poor survival time in both the GSE121931 and PXD021701 cohorts (Figures 5F, I). AUC values for 1-, 2-, and 3-years were 0.630, 0.651, and 0.633 in the GSE121931 cohort, respectively (Figure 5G). AUC values for 1-, 2-, and 3-years were 0.629, 0.620, and 0.600 in the PXD021701 cohort, respectively (Figure 5J). These findings suggested that the five-TrpG signature had good prognostic value in both cohorts. Multivariate Cox regression analysis was used to identify the independent prognostic factor for ESCC patients by incorporating the clinical clinicopathological characteristics (tumor grade and lymph node stages), and the five-TrpG signature, HAAO, ALDH2, and lymph node stages were identified as the independent factors for ESCC to generate a nomogram for 1-, 2-, 3-year survival prediction (Figure 5K). The calibration curve indicated the good performance of the nomogram for 2-year survival prediction (Figure 5L). The decision curves indicated the sensitivity of the factors (Figure 5M). Additionally, we collected ESCC samples to examine the expression levels of five protective factors (MAOA, AKR1A1, ALDH9A1, HAAO, and ALDH2). The results revealed that the expression of MAOA, AKR1A1, ALDH9A1, HAAO, and ALDH2 was significantly downregulated in ESCC tumor tissues compared to non-tumor tissues (Figure 6). These findings suggest that these five genes may serve as potential protective factors and hold promise as diagnostic biomarkers for ESCC.

### 3.5 Correlation analysis between the TrpG risk score and immune cell infiltration, immune checkpoint gene expression, and drug sensitivity

**FIGURE 7:**
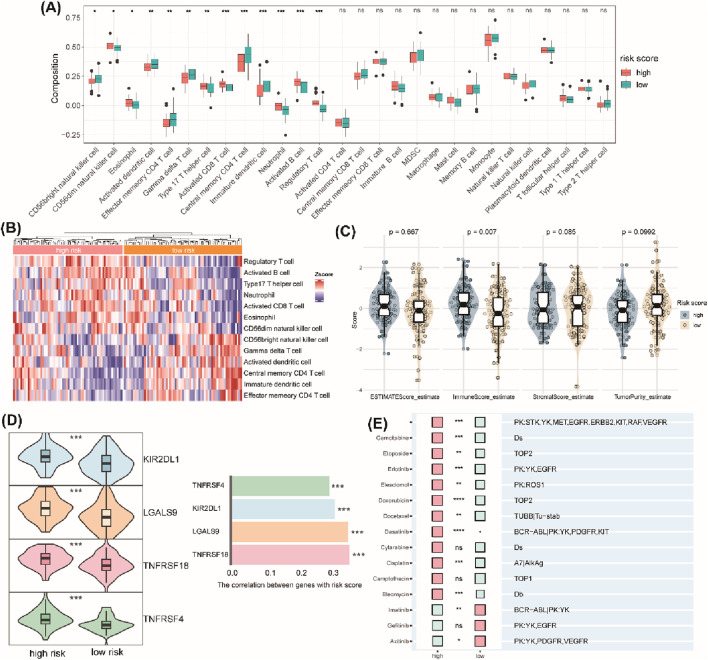
Correlation analysis between the TrpG risk score and immune cell infiltration, immune checkpoint gene expression, and drug sensitivity. **(A)** Boxplots show the differences in infiltrated immune cells between the high-risk and low-risk groups. **(B)** The heatmap showing the infiltrated immune cells between the high-risk and low-risk groups. **(C)** Violin plots of the differences in the ESTIMATE score, immune score, stromal score, and tumor purity between the high-risk and low-risk groups. **(D)** Left: Violin plots of the differences in the expression of immune checkpoints (KIR2DL1, LGALS9, TNFRSF18, TNFRSF4) between the high-risk and low-risk groups. Right: Histogram of the correlation between the expression of immune checkpoints and risk score. **(E)** Histogram of the response to drug therapy between the high-risk and low-risk groups.

We next evaluated the correlation between the TrpG risk score, immune characteristics, and drug sensitivity. The results indicated that the high enrichment scores of regulatory T cells (Treg), CD56 dim natural killer (NK) cells, eosinophils, type 17 T helper (Th17) cells, activated CD8 T cells, neutrophils, and activated B cells were significantly enriched in the high-TrpG risk score group compared with the low-TrpG risk score group. However, CD56 bright (NK) cells, activated dendritic cells (DCs), effector memory CD4 T cells, gamma delta (γδ) T cells, central memory CD4 T cells, and immature DCs were significantly decreased in the high-TrpG risk score group compared with the low-TrpG risk score group (Figures 7A, B). We also found a high immune score in the high-TrpG risk score group compared with the low-TrpG risk score group (Figure 7C). Moreover, we found high expression of immune checkpoint genes (KIR2DL1, LGALS9, TNFRSF18, and TNFRSF4) in the high-TrpG risk score group compared with the low-TrpG risk score group (Figure 7D). The above findings suggest that the TrpG risk score is positively associated with immune cell infiltration; a high TrpG risk score possibly indicates more immune activation. We also estimated the sensitivity to drug therapy, resulting in ESCC patients with high-TrpG risk scores being less sensitive to drugs, such as lapatinib, gemcitabine, etoposide, erlotinib, elesclomol, doxorubicin, docetaxel, dasatinib, cisplatin, and bleomycin; however, they were more sensitive to imatinib and axitinib (Figure 7E).

### 3.6 Correlation analysis between the TrpG risk score and microbial abundance

**FIGURE 8:**
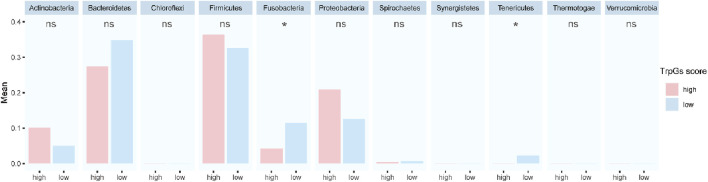
Correlation analysis between the TrpG risk score and microbial abundance.

Furthermore, to explore whether there were specific candidate microbial abundances associated with the TrpG risk score. As shown in Figure 8, we discovered that the relative abundance of *Fusobacteria* and *Tenericutes* was decreased in the high-TrpG risk score group compared to the low-TrpG risk score group. These findings indicate that microbes may be involved in the progression of ESCC.

## 4 Discussion

Growing evidence highlights the vital role of tryptophan (Trp) metabolism in esophageal squamous cell carcinoma (ESCC), particularly involving key enzymes such as IDO1 and TDO2. Elevated IDO1 expression is associated with unfavorable clinical outcomes in ESCC patients (Kiyozumi et al., 2019). Similarly, TDO2 overexpression correlates with tumor growth, progression, immune regulation, and poor outcomes in ESCC patients (Pham et al., 2018; Zhao et al., 2021). Nevertheless, many Trp metabolism-related markers in ESCC remain unidentified.

In the present study, transcriptome analysis identified 28 DE-TrpGs in ESCC compared with normal samples, including eight upregulated (STAT1, IL4I1, TDO2, NMNAT3, KMO, IDO1, OGDHL, and ACAT2) and 20 downregulated DE-TrpGs (ALDH9A1, ALDH3A2, ADH1B, AOX1, MAOA, ECHS1, ALDH2, ACAT1, HAAO, ALDH7A1, HADHA, MAOB, AKR1A1, CYP4X1, INMT, CYP2U1, CAT, HADH, NMNAT1, and UGT2B11). Meanwhile, based on the scRNA-seq data, eight primary cell populations were identified in ESCC: T cells, mast cells, NK cells, myeloid cells, fibroblasts, endothelial cells, B cells, and epithelial cells. Of these cell populations, the population of fibroblasts with the highest TrpG score was identified in the TME of ESCC. Notably, 18 of the 20 downregulated DE-TrpGs (ALDH9A1, ALDH3A2, ADH1B, AOX1, MAOA, ECHS1, ALDH2, ACAT1, HAAO, ALDH7A1, HADHA, AKR1A1, CYP4X1, INMT, CYP2U1, CAT, HADH, and NMNAT1) showed significantly reduced expression specifically in ESCC fibroblasts compared to their normal counterparts. Consequently, we focused our analysis on fibroblasts and further defined them as four distinct subtypes, including normal fibroblasts, myofibroblasts, antigen_CAFs, and perivascular‐like. We also identified malignant epithelial cells and observed altered communication between these malignant cells and either normal fibroblasts or myofibroblasts within the ESCC TME. This intercellular crosstalk appears to be mediated through several signaling pathways, including LAMININ, HSPG, ARGN, MK, PTN, EGF, and IGF.

Cell-to-cell communication within the TME critically influences tumor progression. A feedback loop between lung fibroblasts and lung cancer cells has been identified in lung cancer, with the feedback loop maintained by Trp metabolism to sustain lung cancer progression (Hsu et al., 2016). It has also been found that fibroblasts release a tryptophan metabolite that blocks cancer cell epithelial-mesenchymal transition (EMT), migration, invasion, and metastasis (Cheng et al., 2016). A scRNA-sequencing analysis indicated that TDO2+ myofibroblasts distant from the tumor nest induce the transformation of CD4^+^ T cells into Tregs and cause CD8^+^ T cell dysfunction in oral squamous cell carcinoma (OSCC) (Hu et al., 2022). IDO1+ ovarian cancer (OC) cells were found to be mediated by exosomes to promote endothelial cell mitophagy (Ying et al., 2024). Building on this evidence on the role of Trp metabolism in intercellular crosstalk, we reported for the first time that communication between malignant epithelial cells and normal fibroblasts or myofibroblasts is dysregulated by Trp metabolism in ESCC.

Therefore, we explored the prognostic values of the 18 DE-TrpGs that were downregulated in tumor samples compared with normal samples using either bulk RNA-seq data or scRNA-seq data. Five TrpGs (MAOA, AKR1A1, ALDH9A1, HAAO, ALDH2) were identified and validated as the protective factors for ESCC by machine learning. Consistent with this finding, analysis of clinical samples confirmed significantly downregulated expression of MAOA, AKR1A1, ALDH9A1, HAAO, and ALDH2 in ESCC tumor tissues compared to normal tissues.

Monoamine oxidase A (MAOA), primarily known as a mitochondrial enzyme in the brain (Kolla and Bortolato, 2020) and as an immune checkpoint in antitumor therapy (Wang et al., 2021), also negatively regulates the Trp metabolism-mediated anti-ferroptotic pathways that promote tumor growth (Liu et al., 2023). Consistent with this role, we found that MAOA acts as a protective factor in ESCC by influencing the Trp metabolism. Aldehyde reductase (AKR1A1) is a novel mammalian S-nitroso-glutathione reductase (SNO-CoA) (Stomberski et al., 2019).

The polymorphic allele C of the AKR1A1 rs2088102 might act as a potential protective factor for chemotherapy in breast cancer patients (Cui et al., 2021). Here, we first reported the protective role of AKR1A1 in ESCC, mediated by Trp metabolism regulation. Aldehyde dehydrogenase 9 family member A1 (ALDH9A1) and aldehyde dehydrogenase 2 (ALDH2) belong to the aldehyde dehydrogenase (ALDH) superfamily, catalyzing aldehyde oxidation to carboxylic acids using NAD(P)+ as a coenzyme (Suazo et al., 2023; Li et al., 2016; J et al., 2019). The role of ALDH9A1 in tumors is rarely reported, with susceptibility loci of ALDH9A1 for renal cell carcinoma (RCC) and prostate cancer (Henrion et al., 2015; Bova et al., 2016). Our study newly identified a potential function for ALDH9A1 in ESCC. Conversely, ALDH2 contributes to the occurrence, progression, and treatment of various types of cancer and acts as a potential therapeutic target for cancer therapy (Zhang and Fu, 2021; Yao et al., 2021). Genetic polymorphisms of ALDH2 are associated with the risk of ESC in China (Yang et al., 2007; Wu et al., 2013).

3‐Hydroxyanthranilate 3,4‐dioxygenase (HAAO) encodes the enzymes essential for the NAD + *de novo* synthesis pathway (Schüle et al., 2021). Methylation of HAAO serves as a valuable prognostic marker in ovarian cancer (OC) and prostate cancer (PC) (Huang et al., 2009; Litovkin et al., 2014; Li Y. et al., 2021), and loss of HAAO promotes cancer cells resistant to ferroptosis (Liu et al., 2023). We were the first to identify HAAO as a valuable prognostic marker for ESCC.

We constructed a TrpG risk score to predict ESCC prognosis. Critically, ESCC patients with high-risk scores exhibited significantly poorer survival outcomes. Our analysis revealed distinct patterns of immune cell infiltration associated with TrpG risk scores. High-risk scores showed increased levels of Treg, CD56dim NK cells, eosinophils, Th17 cells, activated CD8 T cells, neutrophils, and activated B cells. Conversely, the high-risk score displayed reduced levels of CD56bright NK cells, activated DC, effector memory CD4 T cells, γδ T cells, central memory CD4 T cells, and immature DCs. Furthermore, the TrpG high-risk group demonstrated significantly higher expression of the immune checkpoint molecules (KIR2DL1, LGALS9, TNFRSF18, TNFRSF4). Given these distinctly different immune profiles between the TrpG high-risk and low-risk groups, we investigated correlations with drug therapy sensitivity. ESCC patients with high-TrpG risk scores are more sensitive to imatinib and axitinib than those with low-TrpG risk scores. Finally, microbiome analysis indicated that the relative abundance of the bacterial phyla *Fusobacteria* and *Tenericutes* is associated with low-TrpG risk scores.

## 5 Conclusion

In the present study, we integrated multi-omics data, including single-cell RNA sequencing (scRNA-seq), bulk RNA-seq, transcriptomics, proteomics, and microbial abundance profiles, to systematically identify fibroblasts critically involved in tryptophan (Trp) metabolism within ESCC. We identified five key Trp metabolism-related genes (TrpGs) and leveraged these to construct a robust five-gene prognostic risk model for ESCC patients.

## Data Availability

The datasets presented in this study can be found in online repositories. The names of the repository/repositories and accession number(s) can be found in the article/Supplementary Material.
